# Application of the Antibody-Inducing Activity of Glycosphingolipids to Human Diseases

**DOI:** 10.3390/ijms22073776

**Published:** 2021-04-06

**Authors:** Tetsuya Okuda

**Affiliations:** Bioproduction Research Institute, National Institute of Advanced Industrial Science and Technology (AIST) Central 6, 1-1-1 Higashi, Tsukuba 305-8566, Japan; t-okuda@aist.go.jp; Tel.: +81-50-3648-6162

**Keywords:** glycosphingolipids, immune response, anti-glycan antibody, very-long-chain fatty acid, glycoprotein

## Abstract

Glycosphingolipids (GSLs) are composed of a mono-, di-, or oligosaccharide and a ceramide and function as constituents of cell membranes. Various molecular species of GSLs have been identified in mammalian cells due to differences in the structures of oligosaccharides. The oligosaccharide structure can vary depending on cell lineage, differentiation stage, and pathology; this property can be used as a cell identification marker. Furthermore, GSLs are involved in various aspects of the immune response, such as cytokine production, immune signaling, migration of immune cells, and antibody production. GSLs containing certain structures exhibit strong immunogenicity in immunized animals and promote the production of anti-GSL antibodies. By exploiting this property, it is possible to generate antibodies that recognize the fine oligosaccharide structure of specific GSLs or glycoproteins. In our study using artificially synthesized GSLs (artGSLs), we found that several structural features are correlated with the antibody-inducing activity of GSLs. Based on these findings, we designed artGSLs that efficiently induce the production of antibodies accompanied by class switching and developed several antibodies that recognize not only certain glycan structures of GSLs but also those of glycoproteins. This review comprehensively introduces the immune activities of GSLs and their application as pharmaceuticals.

## 1. Introduction

Glycosphingolipids (GSLs) are cell membrane components composed of a mono-, di-, or oligosaccharide and a ceramide. Various molecular species defined by differences in the oligosaccharide structure have been identified in mammalian cells/tissues [1]. These differences in the oligosaccharide structure were found to be indicative of cell lineage, differentiation status, and certain pathologic processes. Due to these function-related differences in oligosaccharide structure, GSLs can be used as cell identification markers. Indeed, several anti-GSL antibodies that specifically recognize certain glycan structures of GSLs have contributed to stem cell research and the diagnosis of various cancers [2].

Pioneering studies in this area revealed that GSLs are immunogenic substances that function as blood group [3] and cancer-associated antigens [2]. A number of antibodies isolated from animals immunized with cancer cells were shown to react specifically with GSLs expressed by those cells, leading researchers to recognize that GSLs are immunogenic substances. Recent studies also revealed that the immunoglobulin repertoire of healthy humans contains various anti-GSL antibodies [4,5], indicating that endogenous GSLs function as antigens and induce B cells to produce antibodies. Although it is poorly understood why mammalian immune cells produce antibodies against GSLs, which then become self-antigens, a number of antibodies that recognize GSLs have been isolated by exploiting this property.

Several immunization methods have been developed in order to efficiently generate anti-GSL antibodies [6,7,8]. However, as these methods do not enable extensive control of the properties of the antibodies induced, such as their epitope affinity and specificity and class/subclass, there is considerable room for significant improvement in anti-GSL antibody technology. A deeper understanding of the mechanism of GSL recognition by the mammalian immune system is needed for the development of high-performance anti-GSL antibodies and the future use of these antibodies as pharmaceuticals.

We previously found that GSLs produced by vascular endothelial cells (ECs) under conditions of inflammation exhibit strong antibody-inducing activity [9,10]. The primary feature of these GSLs is that they contain C24 fatty acids [11,12]. A model study using artificially synthesized GSLs (artGSLs) containing ceramide mimetics with various structures demonstrated that the length of the fatty acid is positively correlated with the immunogenic potential of the GSL [13]. GSLs containing C24 fatty acids exhibit thymus-independent type 2 (TI-2) antigen-like properties, which promotes class switching to IgG3 of antibodies induced in immunized mice [14]. Further analyses showed that the oligosaccharide structure of the GSLs strongly affects class switching of induced antibodies [15]. Using artGSLs that were designed based on these structural characteristics, we developed a method to efficiently induce the production of antibodies that recognize not only the glycans of GSLs as an epitope but also the same glycans on glycoproteins [13].

In addition to their antibody-inducing properties, GSLs are involved in various aspects of the immune response, such as cytokine production, immune signaling, and recruitment of lymphocytes to sites of injury. This review provides a comprehensive introduction to recent studies related to GSLs and the immune response and potential medical applications of GSLs, with a particular focus on our findings.

## 2. Structures and Cell Type/Tissue Distributions of Molecular Species of Mammalian GSLs

**Figure 1:**
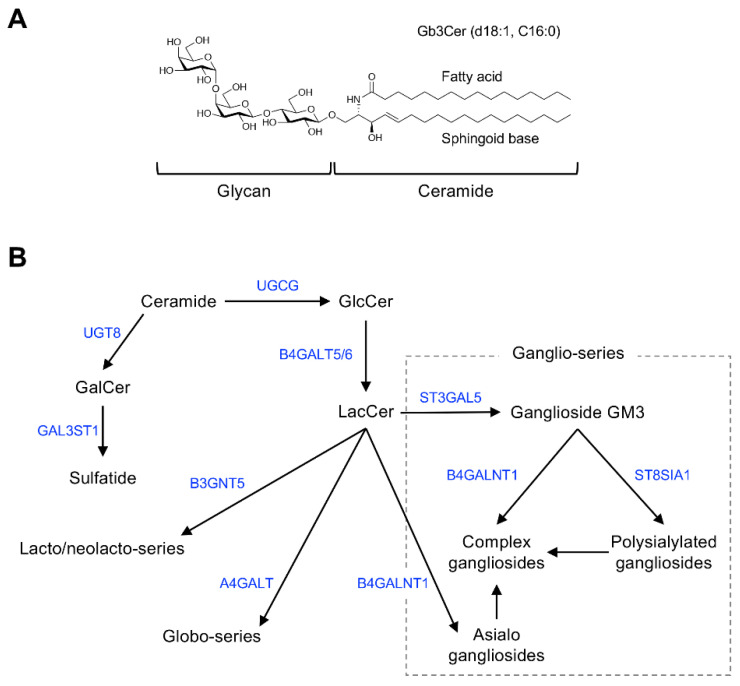
Chemical structure and mammalian glycosphingolipid (GSL) biosynthetic pathway. (**A**) Chemical structure of a typical mammalian GSL, globotriaosylceramide (Gb3Cer), shown as an example. (**B**) Arrows and blue font indicate biosynthetic pathway and catalytic enzymes, respectively. Abbreviations: GlcCer, Glcβ1,1Cer; GalCer, Galβ1,1Cer; LacCer, Galβ1,4Glcβ1,1Cer; GM3, Siaα2,3Galβ1,4Glcβ1,1Cer; UGCG, UDP-glucose ceramide glucosyltransferase; UGT8, UDP glycosyltransferase 8; GAL3ST1, galactose-3-*O*-sulfotransferase 1; B4GALT5/6, β-1,4-galactosyltransferase 5/6; B3GNT5, UDP-GlcNAc: βGal β-1,3-*N*-acetylglucosaminyltransferase 5; A4GALT, α-1,4-galactosyltransferase; B4GALNT1, β-1,4-*N*-acetyl-galactosaminyltransferase 1; ST3GAL5, ST3 β-galactoside α-2,3-sialyltransferase 5; ST8SIA1, ST8 α-*N*-acetyl-neuraminide α-2,8-sialyltransferase 1.

Figure 1 shows the general structure of mammalian GSLs, and Table 1 shows major examples of GSLs identified as cell-surface antigens.

GSLs are glycoconjugates in which a ceramide (*N*-acylsphingosine) is glycosidically bound to a mono-, di-, or oligosaccharide glycan. The glycan moiety is located outside of the plasma membrane of the cell due to its hydrophilicity.

The molecular species of GSLs are classified based on their glycan structure [26]. For example, GSLs containing sialic acid are defined as gangliosides. Ceramide structures are also diverse, and the expression of particular GSL molecular species of ceramides is cell type and tissue specific. Monosialoganglioside GM3 (Figure 1B), the principal initiation structure of gangliosides of the ganglio-series, is widely expressed in mammalian cells and tissues. Complex gangliosides, which are synthesized by elongation of the glycan in GM3, are found primarily in nervous system tissues [26,27]. Galactosylceramide (GalCer/cerebroside) and sulfated GalCer (sulfatide) are also abundantly expressed in nervous system tissues as components of myelin [28].

Globo-series GSLs are expressed primarily in blood cells/vessels and the kidney, lung, and intestine, but their abundance is low in nervous system tissues [29,30]. Stage-specific embryonic antigen (SSEA)-3 and -4, expressed specifically in undifferentiated cells such as induced pluripotent stem cells, are also globo-series GSLs (Gb5Cer and sialyl-Gb5Cer, respectively) [18]. GSLs containing sialyl-Lewis^X^, sialyl-Lewis^a^, and VIM-2 glycans are classified as lacto/neolacto-series GSLs [31,32]. These glycans function as adhesion molecules on the surface of lymphocytes or cancer cells [31,32,33]. The lacto/neolacto-series GSLs expressed in human erythrocytes are blood group substances composed of ABO(H) blood group–type glycans [3,34]. Globo-series GSLs, also known as P blood group substances, are the major GSLs in human erythrocytes [11,17]. Rodent natural killer cells, which specifically express asialo-GM1, can be eliminated by administering anti–asialo-GM1 antibodies to the animals [24,25].

The molecular species of GSLs expressed in mammalian cells vary according to cell differentiation status and pathologic processes. Undifferentiated cells such as stem cells specifically express globo-series GSLs such as SSEA-3 and SSEA-4, whereas these GSLs disappear during differentiation of the cells into somatic cells, which then express other type of GSLs [18]. The type of GSL expressed in a given type of cell or tissues can also differ depending on species. For example, murine stem cells express SSEA-1 (a GSL containing Lewis^X^ glycan) [35,36], whereas this GSL antigen is not expressed by human stem cells [18].

Malignant transformation of cells is associated with structural alterations in GSL glycans; cancer cells express different GSLs than their parent cells [2]. For example, disialylated ganglioside GD3 is expressed in melanoma cells [23], Gb3Cer/CD77 is expressed in Burkitt lymphoma cells [16], and globo-H is expressed in breast cancer cells [19,20]. Thus, these GSLs can be monitored as cancer-specific antigens.

A monoclonal antibody that recognizes sialyl-Lewis^a^ as the epitope, NS19-9, was generated by immunizing mice with SW1116 colorectal cancer cells, which specifically express GSLs with sialyl-Lewis^a^ glycan [21]. The NS19-9 antibody can be used to detect a serum glycoprotein containing sialyl-Lewis^a^, which has been identified as a useful diagnostic marker (CA19-9) for gastrointestinal cancers [22]. In addition to NS19-9, a number of monoclonal antibodies that specifically recognize certain GSL glycan structures as epitopes have been generated by immunizing host animals with various types of cancer cells. These studies led researchers to realize that GSLs are immunogenic substances.

## 3. Role of GSLs in the Mammalian Immune Response

Recent research findings indicate that GSLs are involved in various aspects of the immune response in mammals. Table 2 summarizes the primary findings.

**Table 2:** Functions of GSLs in mammalian immune cells.

GSL	Target or Expressing Cells	Function	Ref.
αGalCer	NKT cells	Stimulate cytokine production	[37]
VIM-2/sLe^X^-terminated GSLs	Human neutrophils, other lymphocytes	Recruitment of lymphocytes to sites of injury	[32,38]
CD77/Gb3Cer	B cells	Regulate apoptosis	[16]
LacCer	Neutrophils	Regulate signal transduction	[39]
Gangliosides	T cells	Regulate T-cell maturation	[40]
Gb4Cer, GM3	ECs, adipocytes, monocytes/macrophages	Regulate TLR4 signaling	[41,42,43]
Gb4Cer	ECs	Induction of anti-Gb4Cer/Gb3Cer antibody production	[9]

Well-characterized GSL functions in mammalian immune cells are shown. Abbreviations: ECs, vascular endothelial cells; NKT, natural killer T; TLR4, Toll-like receptor 4.

α-linked monosaccharyl ceramides such as α-galactosylceramide (αGalCer), isolated from the marine sponge Agelas mauritianus and Sphingomonas bacteria, activate mammalian natural killer T (NKT) cells and promote cytokine production [37,44,45,46,47,48]. αGalCer forms a complex with the CD1d molecule of antigen-presenting cells, which stimulates NKT cells and induces them to produce cytokines [37]. NKT cells activated via this mechanism produce various cytokines, such as IL-4 and IFN-γ, and induce both immunostimulatory and immunosuppressive reactions. Additional research found that isoglobotriaosylceramide (iGb3), which is present in rodent cells/tissues, has an effect similar to αGalCer [49]. In non-human mammals, endogenous ligands for NKT cells, such as iGb3, function in the development of these cells. However, iGb3 is not found in humans [50], and GSLs that function as endogenous ligands for NKT cells have not been clearly identified.

Sialyl-Lewis^X^, VIM-2, sialyl-Lewis^a^, and other glycans with similar structures are present as glycoproteins and GSLs on the surface of lymphocytes [32,33,38,51]. These glycans function as ligands for selectins that are expressed in ECs during inflammation. Adhesion of these glycans and selectins mediates the recruitment of lymphocytes to sites of injury. In some cell types, such as human neutrophils and SW1116 human colon cancer cells, only GSLs function as carriers of these selectin ligand glycans [21,32].

Mangeney et al. found that a globo-series GSL, Gb3Cer/CD77, is a marker for germinal center B cells that induce programmed cell death [16]. Gb3Cer/CD77 is also highly expressed in germinal center B-cell-derived cancer Burkitt lymphoma cells. Taga and Tétaud et al. showed that treatment of these cells with antibodies or verotoxins that specifically recognize Gb3Cer induces apoptosis [52,53]. These results suggest that Gb3Cer/CD77 plays a role in regulating programmed cell death associated with B cell maturation and malignant transformation.

Nakayama et al. revealed that a LacCer-abundant microdomain is found in the plasma and granular membranes of human neutrophils and that this domain mediates neutrophil recognition of β-glucan and lipoarabinomannan expressed by pathogenic bacteria and activates innate immune responses [39]. LacCer is thought to regulate the recognition of these pathogen-derived glycans, and binding of the pathogen to LacCer induces neutrophil responses such as chemotaxis, phagocytosis, and phagolysosome formation via signal transduction involving Src family kinases. In contrast, Nagafuku et al. reported that gangliosides expressed by T cells affect signal transduction via T-cell receptors [40].

Toll-like receptor 4 (TLR4) is a cellular receptor that recognizes lipopolysaccharide (LPS), a component of the cell wall of Gram-negative bacteria [54]. TLR4 plays a fundamental role in pathogen recognition and subsequent activation of innate immune responses, such as inflammation. Kondo et al. found that EC-expressed Gb4Cer attenuates LPS-TLR4 signal transduction by inhibiting the binding of LPS to TLR4 [41]. Nitta et al. reported that renal inflammation mediated via TLR4 signaling is enhanced by Gb4Cer [42]. Kanoh et al. also showed that GM3 affects TLR4 signaling in adipocytes and monocytes and that the effect depends on the fatty acid structure of GM3 [43]. The effect of GSLs on TLR4 signaling appears to depend on the combination of cell type and the GSL molecular species.

**Figure 2:**
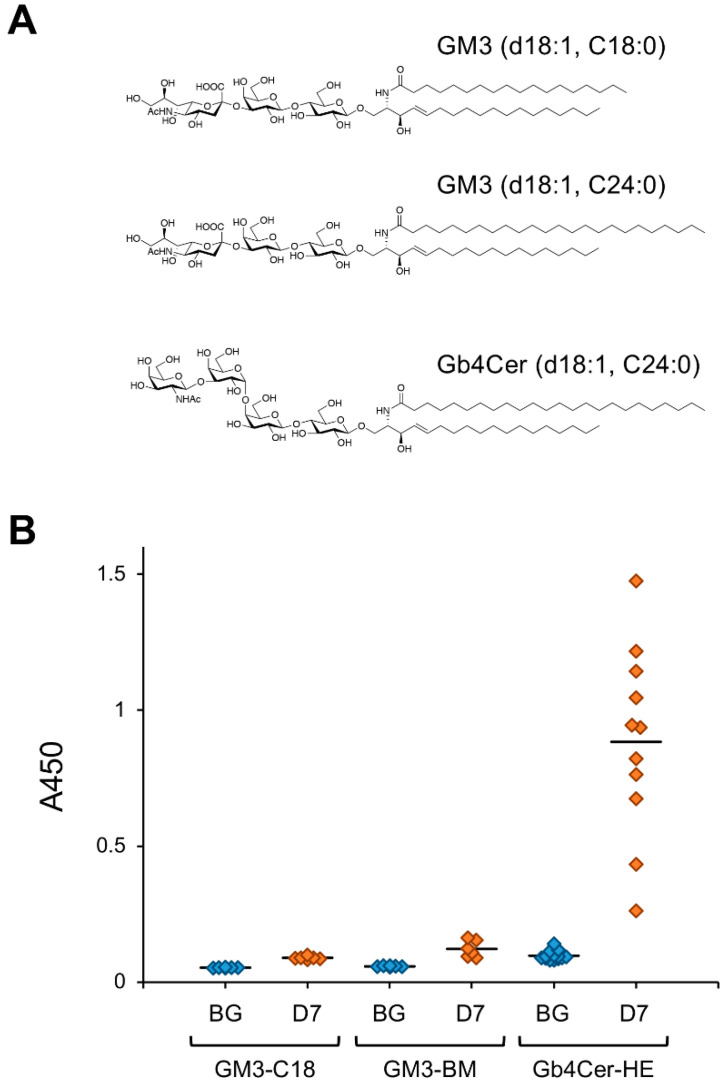
Antibody-inducing activity of mammalian GSLs. (**A**) Chemical structures of GSLs used for immunization experiments. GM3 containing C18:0 stearic acid (GM3-C18) was prepared by chemical synthesis to be a uniform structure. Bovine milk-derived GM3 (GM3-BM) and human erythrocyte-derived Gb4Cer (Gb4Cer-HE) predominantly contain very-long-chain fatty acids with 22–24 carbons. (**B**) Reactivity of mice serum IgMs against immunizing GSL. Mice were immunized with each GSL, and serum samples were prepared 7 days after immunization, as described previously [15]. Reactivity of serum antibodies against immunizing GSLs was analyzed by ELISA (A450). Abbreviations; BG, serum from untreated mice; D7, serum prepared from mice immunized with each GSL 7 days after immunization. Diamonds indicate individual mouse serum samples (n = 6–11). Solid lines indicate average reactivity of serum samples. Values include previously reported data [10].

Stimulation of vascular ECs with the inflammatory mediator tumor necrosis factor–α (TNFα) increases intracellular GSL expression via transcriptional regulation of related genes [11,12,55,56]. Detailed analyses of GSL structures in TNFα-stimulated human umbilical vein ECs revealed that Gb4Cer containing C24 fatty acids is the major component of induced GSLs. We investigated the biological activity of these GSLs and found that Gb4Cer containing C24 fatty acids strongly induces antibody production in mice [9,10]. Figure 2 shows the reactivity to GSLs of serum antibodies of mice immunized with GM3 and human erythrocyte-derived Gb4Cer, which predominantly contains very-long-chain fatty acids with 22–24 carbons [57].

Induction of antibodies against Gb4Cer containing C24 fatty acids was stronger than that of antibodies against GM3, the major GSL widely expressed in mammalian cells and tissues. Due to this strong antibody-inducing activity, mice immunized with Gb4Cer containing C24 fatty acids produce a variety of antibodies, such as anti-Gb4Cer IgG3 and antibodies that react with Gb3Cer, the precursor of Gb4Cer [9]. Saccharide antigens generally induce the production of IgM without class switching, whereas those with strong immunogenicity induce class switching to IgG3 in mice. These IgG3-inducing saccharide antigens are known as TI-2 antigens [14]. Our findings indicate that Gb4Cer containing C24 fatty acids are TI-2 antigens. Recent studies using immunoglobulin preparations revealed that human lymphocytes constitutively produce antibodies that react with Gb4Cer and its derivatives [4,5]. These antibodies are thought to play a role in host defense because Gb4Cer and its derivatives serve as host cell attachment sites for pathogenic bacteria, bacterial toxins, and viruses [4]. As pathogen infection induces inflammatory responses in ECs, we speculate that inflammation-induced cellular expression of Gb4Cer containing C24 fatty acids and its derivatives promotes the production of antibodies specific to Gb4Cer and its derivatives. These induced antibodies are suspected to in turn function in host defense against pathogen infection.

## 4. Antibody-Inducing Activity of GSLs

As the structure of GSLs is highly conserved across animal species, antibodies that react with them become autoantibodies. The expression of autoantibodies can lead to the development of autoimmune disease in the host; thus, the B lymphocytes that produce these antibodies typically disappear during maturation or become non-responsive to antigens. However, recent studies have shown that healthy humans and mice carry antibodies against GSLs. B lymphocytes of mice immunized with GSLs strongly produce antibodies that react with cellular GSLs as self-antigens, but in most cases, no adverse health effects are observed in these mice. Although it remains unclear why such an immune response would be induced in mammals, it can be exploited to generate anti-GSL antibodies.

Various immunization methods have been established for the efficient production of anti-GSL antibodies in host animals using GSLs adsorbed to acid-treated *Salmonella minnesota* or lipid A–containing liposomes [6,7]. Using these methods, antibodies that specifically recognize the glycan moiety of a GSL as an epitope have been generated. Our recent study demonstrated that both artificially designed GSLs and natural GSLs derived from mammalian cells can be used to generate anti-glycan antibodies by these immunization methods [8,9,10,13,15,58]. By optimizing the structure of an artificial (art) GSL, efficient generation of antibodies that recognize specific glycoprotein glycans can be achieved [8,13,15].

In studies using mice as the host, repetitive immunization with artGSLs efficiently promoted the production of antibodies that specifically recognize the glycan structure of the target artGSL. A variety of antibodies was found to be induced in immunized mice, some of which reacted with glycoproteins containing glycans with the same structures as the GSLs used for immunization [13,15]. The antibody-inducing activity of a GSL depends on its structure. For example, Figure 2 shows the immunogen reactivity of serum antibodies in mice immunized with Gb4Cer derived from human erythrocytes (Gb4Cer-HE) and GM3 containing different fatty acids (GM3-C18 and bovine milk–derived GM3 [GM3-BM]). Gb4Cer derived from human erythrocytes predominantly contains C24 fatty acids, and its structure is associated with strong immunogenicity. Antibodies are induced in immunized mice much more strongly against Gb4Cer than GM3. A weak but similar tendency was observed with GM3-C18, which contains only stearic acid, and GM3-BM, which predominantly contains very-long-chain fatty acids with 22–24 carbons. These results indicate that C24 fatty acids and Gb4Cer-type glycans are correlated with strong antibody-inducing activity of GSLs.

**Figure 3:**
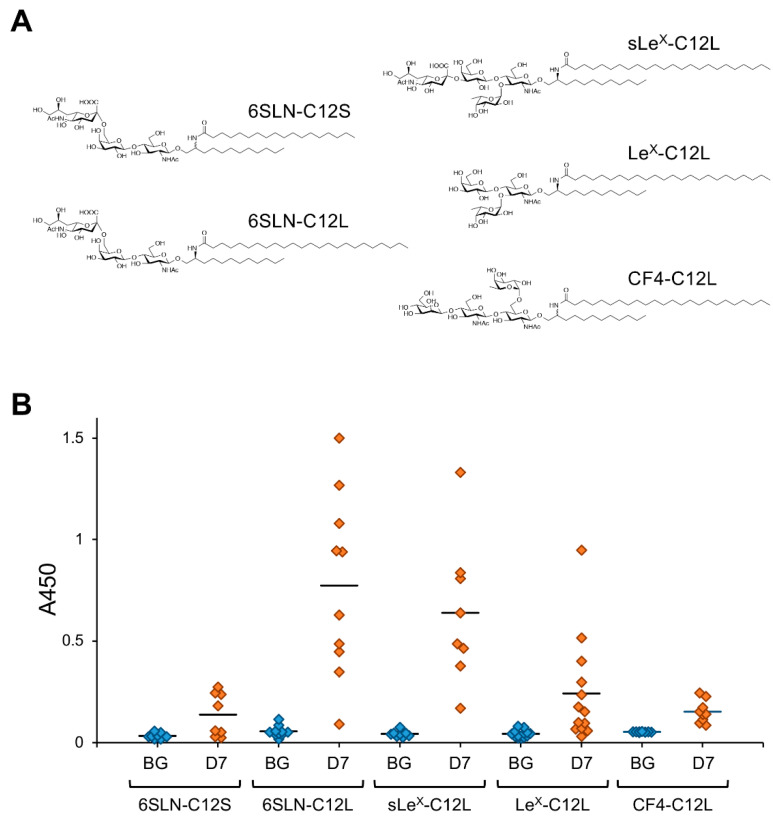
Antibody-inducing activity of artificially synthesized GSLs (artGSLs). (**A**) Chemical structures of artGSLs used for immunization experiments. The ceramide mimetics C12S and C12L are composed of a saturated C12-sphingosine mimetic and stearic acid (C18:0) or lignoceric acid (C24:0), respectively. These ceramide mimetics are bound to the oligosaccharide via a β-linkage. Abbreviations: 6SLN, 6′-sialyl LacNAc/Neu5Acα2,6Galβ1,4GlcNAc; sLe^X^, sialyl Lewis^X^/Neu5Acα2,3Galβ1,4(Fucα1,3)GlcNAc; Le^X^, Lewis^X^/Galβ1,4(Fucα1,3)GlcNAc; CF4, core-fucosylated tetrasaccharide/Manβ1,4GlcNAcβ1,4(Fucα1,6)GlcNAc. (**B**) Reactivity of mice serum IgMs against immunizing GSL. Mice were immunized with each artGSL, and serum samples were prepared 7 days after immunization, as described previously [15]. Reactivity of serum antibodies against immunizing GSLs was analyzed by ELISA (A450). Abbreviations; BG, serum from untreated mice; D7, serum prepared from mice immunized with each GSL 7 days after immunization. Diamonds indicate individual mouse serum samples (n = 8–13). Solid lines indicate average reactivity of serum samples. Values include previously reported data [13,15].

The effect of C24 fatty acids on the antibody-inducing activity of GSLs was also demonstrated in model experiments using artificially synthesized GSLs [13]. In an analysis using artGSLs in which ceramide mimetics with simple alkyl structures were bound to 6SLN trisaccharide as a model (Figure 3), 6SLN-C12L containing C24:0 lignoceric acid induced the production of antibodies against the immunizing artGSL more efficiently than a derivative containing C18:0 stearic acid (6SLN-C12S).

**Figure 4:**
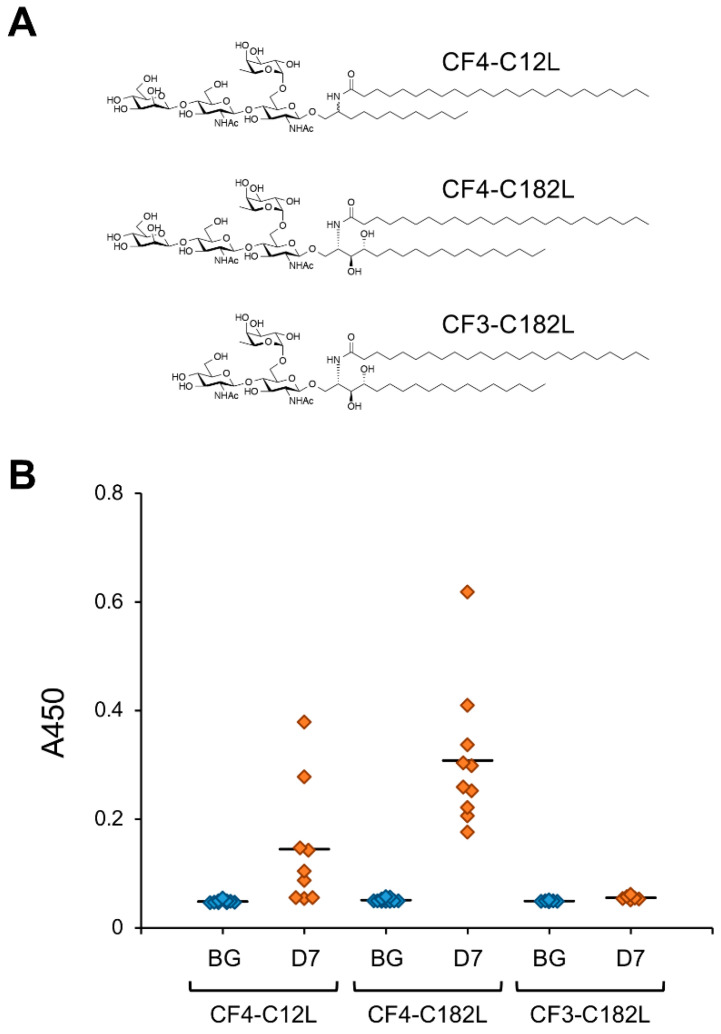
IgG-inducing activity of CF4-C12L and its derivatives. (**A**) Chemical structures of CF4-C12L derivatives used for immunization experiments. The ceramide mimetic C182L is composed of C18-phytosphingosine and lignoceric acid. The ceramide mimetic is bound to the oligosaccharide via a β-linkage. Abbreviations: CF4, core-fucosylated tetrasaccharide/Manβ1,4GlcNAcβ1,4(Fucα1,6)GlcNAc; CF3, core-fucosylated trisaccharide/GlcNAcβ1,4(Fucα1,6)GlcNAc. (**B**) Reactivity of mice serum IgGs against immunizing GSL. Mice were immunized with each artGSL, and serum samples were prepared 7 days after immunization, as described previously [15]. Reactivity of serum antibodies against immunizing GSLs was analyzed by ELISA (A450). Abbreviations; BG, serum from untreated mice; D7, serum prepared from mice immunized with each GSL 7 days after immunization. Diamonds indicate individual mouse serum samples (n = 6–10). Solid lines indicate average reactivity of serum samples. Values include previously reported data [15].

The ceramide mimetic C12L efficiently enhanced the antibody-inducing activity of the corresponding artGSL even when conjugated to glycans with different structures. Although 6SLN conjugated with C12L primarily induces the production of IgM antibodies in mice, it also rapidly induces the production of IgG class antibodies against immunizing artGSLs [13]. Surprisingly, mice immunized with a conjugate of C12L with CF4 tetrasaccharide (which is the structure found in the stem region of glycoproteins with *N*-linked glycosylation) more strongly produced IgG class antibodies compared to mice immunized with other GSLs/artGSLs [15]. In contrast, production of IgM in mice immunized with CF4-C12L was lower than that in mice immunized with other GSLs/artGSLs, indicating that this artGSL efficiently induces class switching in B lymphocytes. Further studies using CF4-C182L, a derivative of CF4-C12L, demonstrated that hydroxylation of the sphingosine portion of C12L enhances the efficiency of IgG induction. However, CF3-C182L, a derivative of CF4-C182L in which the β-mannose of the non-reducing terminal of CF4-C182L has been removed, exhibited minimal antibody-inducing activity (Figure 4).

These results indicate that fine structures of both the oligosaccharide and ceramide portions of artGSLs/GSLs affect their antibody-inducing and class-switching activity.

In mice immunized with artGSLs, antibodies that specifically react with glycoproteins containing the same glycan structure as the immunizing artGSL are induced. As the fine structures of artGSLs affect their antibody-inducing activity, we hypothesize that by optimizing the artGSL structure, a variety of useful anti-glycan antibodies could be produced using this technology.

## 5. Anti-GSL Antibodies in Infectious and Immune Diseases

In the gastrointestinal tract, a wide variety of bacteria and their products such as bacterial toxins interact with the glycoconjugates in mucosal and epithelial cells [59]. For example, the major subtypes of Shiga-like toxin produced by enterohemorrhagic *Escherichia coli* preferentially bind to Gb3Cer and Gb4Cer expressed on the surface of epithelial and endothelial cells, and cause hemorrhagic colitis and hemolytic uremic syndrome characterized by microvascular endothelial damages in the kidney and brain [29,60]. Gb3Cer and Gb4Cer are GSLs characterized by an α-1,4-galactose structure, and it has recently been found that antibodies that recognize α-galactose structures are constitutively produced in humans and mice [4,5,9,61]. As this α-1,4-galactose mediates the interaction of bacteria and their products with the gastrointestinal tract, it is speculated that these antibodies are involved in host defense.

Anti-ganglioside antibodies are associated with the pathophysiology of autoimmune diseases such as Guillain-Barré syndrome and its variant Miller Fisher syndrome [62]. The appearance of these autoantibodies is associated with infection with pathogens such as *Campylobacter jejuni*, cytomegalovirus, Epstein-Barr virus, and *Hemophilus influenzae*. Although insufficient information is available regarding the neurological manifestations of COVID-19, there are many case reports that describe Guillain-Barré syndrome (GBS) as an acute presentation of SARS-CoV-2 [63,64,65,66]. Increased serum titer of anti-ganglioside antibodies was also found in SARS-CoV-2-infected patients with neurological manifestations [66,67].

Anti-ganglioside antibodies are also found in the sera of vaccinated subjects. The vaccination campaign against the H1N1-type influenza A with *Pandemrix* vaccine in several European countries reported a clear increase in narcolepsy cases [62,68]. Although its role in the pathological process is still unclear, anti-ganglioside antibodies, in particular, anti-GM3 and anti-GM4, were found in the sera of 18.1% of patients with *Pandemrix*-induced narcolepsy [68].

Galactose-α-1,3-galactose (α-Gal) is an oligosaccharide that was first described as a cause of immunoglobulin E (IgE)-mediated anaphylaxis in cases of first-in-man reactions to the monoclonal antibody cetuximab [69,70,71,72]. The α-Gal present in mammalian glycoproteins and GSLs, also causes an unusual delayed allergic reaction 3–6 h after ingestion of mammalian meat in individuals with IgE antibodies against α-Gal [70,71,72,73]. As GSLs are slowly digested and absorbed, the GSL form of α-Gal is considered to be associated with this characteristic delay [73].

## 6. Application of Anti-GSL Antibodies to Pharmaceuticals

The antibody-inducing activity of GSLs can be exploited to produce antibodies against various cell surface glycan antigens that serve as cell identification markers. A number of studies examining the development of antibodies using GSLs as immunogens and several methods for generating anti-glycan antibodies have been reported to date.

CA19-9, a cancer-associated serum glycoprotein antigen containing sialyl-Lewis^a^ glycan and used as a diagnostic marker for gastrointestinal cancer, can be detected using the NS19-9 antibody [21]. NS19-9 is a monoclonal IgG1 generated by immunizing mice with SW1116 colon cancer cells that express GSLs containing sialyl-Lewis^a^. The GSL antigens SSEA-3 and SSEA-4, which are widely used as stem cell markers, can be detected using antibodies generated by immunizing with mouse embryos or human teratocarcinoma cells expressing these GSL antigens [74]. In this way, it is possible to generate antibodies that recognize a specific glycan as the epitope via immunization with cells expressing specific GSLs. In contrast, several methods for generating anti-glycan antibodies using neoglycolipid analogues of GSLs as immunogens have also been reported. Ozawa et al. reported that immunizing mice with a neoglycolipid in which an oligosaccharide was reductively conjugated to phosphatidylethanolamine (PE) induced antibodies that recognized glycoconjugates containing this oligosaccharide [75]. The advantage of this method is that neoglycolipids can be easily synthesized by conjugating an oligosaccharide and PE via a simple chemical reaction. Subsequently, Murakami et al. reported an alternative enzymatic method for conjugating oligosaccharides to PE [76]. With the artGSL proposed by us, the induction efficiency and class switching of anti-glycan antibodies can be controlled by optimizing the fine structures of the glycan and ceramide [13,15]. However, as high-performance antibodies generated using these methods are generally IgM class, further research will be required to efficiently generate IgG-class anti-glycan antibodies that are practically useful as bio-pharmaceuticals.

Huang et al. reported that the immunogenicity of globo-H oligosaccharide, a cancer-associated glycan found in a mammalian GSL, can be enhanced by conjugation with protein carriers such as KLH or CRM197 [77]. They reported that these conjugates efficiently induced the production of IgG antibodies that reacted with globo-H and its derivatives in immunized mice. These globo-H conjugates are being considered for use as a vaccine to prevent breast cancer [77,78]. It was also reported that production of IgG-class anti-GSL antibodies can be efficiently induced by immunizing GSL-deficient mice with target GSLs [79,80,81,82,83,84].

Some previously developed anti-GSL IgG antibodies are being applied and studied as antibody therapeutics for the treatment of melanoma and neuroblastoma. Ch14.18 (dinutuximab) and Hu3F8 (naxitamab) are chimeric and humanized IgG antibodies, respectively, that were developed from anti-GD2 IgG3s isolated from mice and specifically recognize ganglioside GD2 [85,86,87]. Dinutuximab has been approved by the US Food and Drug Administration, and Ch14.18 produced in CHO cells (dinutuximab-beta/Qarziba) has been approved by the European Medicines Agency for the treatment of high-risk neuroblastoma. Hu3F8 is also undergoing clinical trials to verify its efficacy. The application of an anti-GD3 chimeric antibody (KW2871/ecromeximab) that specifically reacts with GD3 expressed on malignant melanoma cells is also being investigated [88]. KW2871 was developed based on anti-GD3 IgG3 isolated in mice [89,90].

As IgM antibodies exhibit strong antitumor effects, the use of anti-GSL IgMs for antibody therapeutics has been examined. Clinical trials of the human anti-GM3 IgM L612 [91] are underway for treatment of GM3-positive human melanoma [92,93]. Patients infused with this antibody after surgery were reported to have no side effects or signs of recurrence for 5 years. Furthermore, with the artGSL technology that we are developing, it would be possible to efficiently generate IgM antibodies that specifically recognize the glycan moieties of glycoproteins [13,15]. Such anti-glycan IgMs that react with glycoproteins could also be used as antibody therapeutics. For example, the human monoclonal IgM mAb216, which recognizes glycosylation epitopes on B lymphocytes, is undergoing clinical trials to evaluate its potential for therapeutic application in treating B-cell precursor acute lymphoblastic leukemia [94]. mAb216 binds to B lymphoblasts in patients with acute lymphoblastic leukemia and enhances the effects of drugs such as vincristine and exerts cytotoxicity against cancer cells in conjunction with complement.

We consider anti-glycan IgM a promising candidate for use as a next-generation bio-pharmaceutical and are thus working to develop technologies to better utilize IgM antibodies. In particular, we are trying to promote the application of anti-glycan IgMs as diagnostic agents and pharmaceuticals by establishing purification methods necessary for downstream processes. In our recently established IgM purification method using porous zirconia particles, anti-glycan IgMs can be purified without impairing their antigen-binding activity in a gentle process using phosphate buffers in neutral pH ranges [95]. The porous zirconia particles have pores similar in size to one unit of immunoglobulin, and the surface of the particles is modified with EDTPA, which has a specific affinity for immunoglobulins. These properties make it possible to purify IgM with high purity from antibody preparations containing serum. We have demonstrated that various IgM and IgG clones that specifically recognize glycan epitopes on glycoproteins and GSLs generated by immunizing mice with GSLs or artGSLs can be easily purified without loss of activity using this system.

## 7. Concluding Remarks

Technologies for inducing the production of antibodies that specifically recognize fine structures of glycans could facilitate the development of new diagnostic methods and therapeutic for cancers and infectious diseases related to glycoconjugates. Although previous efforts to develop anti-glycan antibodies have led to the development of several pharmaceuticals, there remains considerable room for improvement in the technology. Furthermore, many details regarding how the mammalian immune system recognizes glycans remain unclear. GSLs have properties that are readily recognized by the mammalian immune system; thus, GSLs are suitable molecules for investigations examining how the immune system recognizes glycans. We believe that findings obtained through fundamental and applied studies of GSLs will contribute substantially to the development of next-generation pharmaceuticals.

## Figures and Tables

**Table 1:** Glycosphingolipid (GSL) markers useful for cell identification.

GSL/Antigen Structure	Major Expressing Cell or Expressing Cancers	Ref.
Gb3Cer/CD77 Galα1,4Galβ1,4Glcβ1,1Cer	Burkitt lymphoma	[16]
Gb4Cer/blood group P antigen GalNAcβ1,3Galα1,4Galβ1,4Glcβ1,1Cer	Erythrocytes	[17]
Gb5Cer/SSEA-3 Galβ1,3GalNAcβ1,3Galα1,4Galβ1,4Glcβ1,1Cer	Stem cells/iPS cells	[18]
Sialyl-Gb5Cer/SSEA-4 Siaα2,3Galβ1,3GalNAcβ1,3Galα1,4Galβ1,4Glcβ1,1Cer	Stem cells/iPS cells	[18]
Fucosyl-Gb5Cer/Globo-H Fucα1,2Galβ1,3GalNAcβ1,3Galα1,4Galβ1,4Glcβ1,1Cer	Breast and other cancers	[19,20]
Sialyl Lewis^a^/CA19-9-terminated GSL Siaα2,3Galβ1,3(Fucα1,4)GlcNAcβ1,3Galβ1,4Glcβ1,1Cer	Gastrointestinal cancer	[21,22]
GD3 Siaα2,8Siaα2,3Galβ1,4Glcβ1,1Cer	Melanoma	[23]
Gg4Cer (asialo-GM1) Galβ1,3GalNAcβ1,4Galβ1,4Glcβ1,1Cer	Natural killer cells	[24,25]

Major GSL antigens used to identify human cells are shown. Abbreviations: Cer, ceramide; Fuc, fucose; Gal, galactose; GalNAc, *N*-acetylgalactosamine; Glc, glucose; GlcNAc, *N*-acetylglucosamine; Sia, sialic acid.

## Data Availability

The datasets generated during and/or analyzed during the current study are available from the corresponding author on reasonable request.
